# Genetic variants of lncRNA HOTAIR contribute to the risk of osteosarcoma

**DOI:** 10.18632/oncotarget.7957

**Published:** 2016-03-07

**Authors:** Quan Zhou, Fengli Chen, Zhongting Fei, Jiali Zhao, Yong Liang, Wei Pan, Xingxiang Liu, Donghui Zheng

**Affiliations:** ^1^ Department of Orthopaedics, Huai'an Hospital Affiliated of Xuzhou Medical College and Huai'an Second Hospital, Huai'an, Jiangsu, China; ^2^ Department of Central Laboratory, Huai'an First People's Hospital, Nanjing Medical University, Huai'an, Jiangsu, China; ^3^ Department of Clinical Laboratory, Huai'an 4th people's Hospital, Huai'an, Jiangsu, China; ^4^ Department of Central Laboratory and Department of Nephrology, Huai'an Hospital Affiliated of Xuzhou Medical College and Huai'an Second Hospital, Huai'an, Jiangsu, China

**Keywords:** osteosarcoma, susceptibility, variant, lncRNA, HOTAIR

## Abstract

Osteosarcoma (OS) is the most common primary malignant bone tumor in adolescents and young adults. However, the essential mechanisms underlying osteosarcomagenesis remain obscure. The HOTAIR, a well-known long noncoding RNA (lncRNA), is involved in pathogenesis and progress of multiple tumors. To reveal the potential role of lncRNA HOTAIR in OS carcinogenesis, we conducted a two-stage, case-control study among Chinese population with 900 OS cases and 900 controls to evaluated associations of its genetic variants with OS risk. We found that C allele of rs7958904 was associated with a significantly decreased OS risk when compared with G allele (OR: 0.77; 95% CI: 0.67-0.90; *P* = 6.77×10-4). Functional analyses on HOTAIR Expression showed that the expression level of HOTAIR in OS tissues was significantly higher than that in corresponding normal tissues, and subjects with the rs7958904 CC genotype had significantly lower HOTAIR RNA levels than those of other genotypes. This should be the first study to examine the association between HOTAIR variants and OS risk.

## INTRODUCTION

Osteosarcoma (OS), which derives from primitive bone-forming mesenchymal cells, is the most common primary bone malignancy and the eighth-most common form of childhood cancer, comprising 2.4% of all malignancies in pediatric patients, and about 20% of all primary bone cancers [1-5]. According to the Surveillance, Epidemiology, and End Results (SEER) program, approximately 7,104 cases of malignant primary OS patients were identified during 1999-2008 in United States, of which 5,379 were appendicular and 1,725 were axial [3]. For many years, accumulated evidences suggested that multiple genetic and environmental factors play pivotal roles in the pathogenesis of OS [6, 7]. However, the essential mechanisms underlying osteosarcomagenesis and progression continue to be obscure [8-10].

Recently, long non-coding RNAs (lncRNAs) have been identified for their wide range of biological regulatory functions in the carcinogenesis and progression of many cancers [11-14]. Loss of HOTAIR, a lncRNA in the mammalian HOXC locus that binds to and targets the PRC2 complex to the HOXD locus, can inhibit cancer invasiveness, particularly in cells that possess excessive PRC2 activity [15]. It has been widely explored for its genetic variants, expression level and carcinogenesis, tumor development and progression [16-19]. Previously, studies have investigated the association between HOTAIR variants and several cancers, such as breast cancer, gastric cancer, colorectal cancer, esophageal squamous cell carcinoma, and gastric cardia adenocarcinoma [17, 20-25]. However, little is known about the role of these gene polymorphisms in the carcinogenesis of OS. Thus, to clarify this association, we analyzed the role of HOTAIR variants in OS Development in a two-stage case-control study among a Chinese population.

## RESULTS

### Characteristics of the study population

In current study, a total of 900 OS cases and 900 controls were recruited in two stages, and there were no significant differences between OS cases and healthy controls for each stage regarding to age and gender (all *P* > 0.05), which indicates that the frequency matching was adequate. The tumors were mainly located at extremities, and half of the cases were metastatic OS. About 39.8% of cases' enneking stage were defined as III in stage 1, and 40.1% in stage 2.

**Table 1:** presents the distribution of selected characteristics among OS cases and controls

	Stage 1			Stage 2		
Category	Cases (*N* = 500)	Controls (*N* = 500)	*P* Value	Cases (*>N* = 400)	Controls (*N* = 400)	***P*Value**
Age (yr)						
Mean ± SD	23.5±8.5	23.3±7.1	0.686	23.7±5.5	23.2±5.2	0.187
Gender						
Male	300 (60.0%)	295 (58.9%)	0.747	237 (59.2%)	238 (59.5%)	0.943
Female	200 (40.0%)	205 (41.1%)		163 (40.8%)	162 (40.5%)	
Tumor location						
Extremities	394 (78.7%)			320 (80.0%)		
Other	106 (21.3%)			80 (20.0%)		
Metastasis						
Yes	238 (47.5%)			207 (51.8%)		
No	262 (52.5%)			193 (48.2%)		
Enneking stage						
I-II	301 (60.2%)			240 (59.9%)		
III	199 (39.8%)			160 (40.1%)		

### Associations of tagSNPs and OS risk

In stage 1, the genotypes distributions of 3 SNPs (rs4759314, rs7958904 and rs874945) among the controls were in accordance with Hardy-Weinberg equilibrium (all *P* > 0.05). The genotype distribution of all the tagSNPs and their associations with OS risk in our Stage 1 are shown in Table 2. SNP rs7958904 and rs874945 showed significant associations with OS risk in log-additive model (*P* value = 0.023, 0.024 respectively). For rs7958904, C allele was associated a significantly decreased OS risk when compared with G allele (OR: 0.79; 95% CI: 0.65-0.97); while for rs874945, A allele was associated a significantly increased OS risk when compared with G allele (OR: 1.28; 95% CI: 1.03-1.59). No notable associations between rs4759314 and OS risk in the Stage 1 were observed. Therefore, we validated the effects of rs874945 and rs7958904 in the Stage 2, to further confirm the association observed in the first stage. As shown in Table 2, the association of rs874945 disappeared in the Stage 2 (*P*_trend_ = 0.614). However, the protective effects of rs7958904 still existed in the Stage 2 (*P*_trend_ = 0.011). When combined together, C allele was significantly associated with a decreased OS risk when compared with G allele (OR: 0.77; 95% CI: 0.67-0.90; *P* = 6.77×10^−4^). The adjusted OR for the carriers with the CG genotype was 0.82 (95% CI: 0.67-1.00) and for those with the CC genotype was 0.57 (95% CI: 0.40-0.81) compared with the GG genotype. We also conducted stratified analyses by age, gender, and tumor location (Table 3). The results didn't change materially.

**Table 2:** Genetic variants of HOTAIR and Osteosarcoma risk

Stage	Genotype	Cases	Controls	Adjusted OR (95% CI)*
**rs4759314**	AA	423	425	1.00 (reference)
	AG	62	64	0.97 (0.67-1.42)
	GG	15	11	1.37 (0.62-3.01)
	G vs A			1.08 (0.79-1.47)
	P trend			0.638
**rs874945**				
**Stage 1**	GG	310	338	1.00 (reference)
	AG	150	135	1.21 (0.92-1.60)
	AA	40	27	1.62 (0.97-2.68)
	A vs G			1.28 (1.03-1.59)
	P trend			**0.024**
**Stage 2**	GG	267	270	1.00 (reference)
	AG	106	108	0.99 (0.72-1.36)
	AA	27	22	1.24 (0.69-2.23)
	A vs G			1.07 (0.83-1.36)
	P trend			0.614
**rs7958904**				
**Stage 1**	GG	295	266	1.00 (reference)
	CG	180	194	0.83 (0.64-1.09)
	CC	25	40	0.56 (0.33-0.95)
	C vs G			0.79 (0.65-0.97)
	P trend			**0.023**
**Stage 2**	GG	229	200	1.00 (reference)
	CG	140	152	0.80 (0.60-1.08)
	CC	31	48	0.56 (0.35-0.92)
	C vs G			0.75 (0.60-0.93)
	P trend			**0.011**
**Combined results**	GG	524	466	1.00 (reference)
	CG	320	346	0.82 (0.67-1.00)
	CC	56	88	0.57 (0.40-0.81)
	C vs G			0.77 (0.67-0.90)
	P trend			**6.77×10^−4^**

* Adjusting for age, and gender

### Functional relevance of rs7958904 on HOTAIR expression

**Figure 1:**
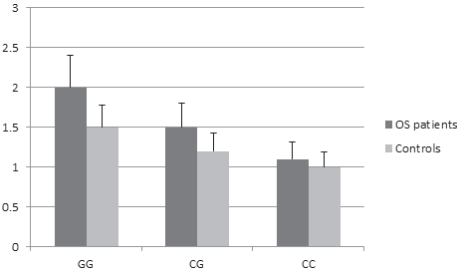
HOTAIR RNA expression (mean ± SD) in normal and OS tissues grouped by SNP rs7958904 of HOTAIR

To confirm the abnormal expression of HOTAIR in OS patients, we evaluated the HOTAIR levels in 100 paired tissues of OS patients and corresponding normal tissues. As shown in Figure 1, the expression level of HOTAIR in OS tissues was significantly higher than that in corresponding normal tissues (*P* = 0.008). Subjects with the rs7958904 CC genotype had significantly lower HOTAIR RNA levels (mean ± SD) than those with the GG genotypes in normal and OS tissues (*P* < 0.01).

**Table 3:** HOTAIR rs7958904 and Osteosarcoma risk stratified by co-variables

**Variables**	**Categories**	**Genotype**	**Adjusted OR (95% CI)**	**P trend**
Gender				
	Male	GG	1.00 (reference)	
		CG	0.81 (0.63-1.03)	
		CC	0.55 (0.39-0.78)	
		C vs G	0.79 (0.66-0.94)	**0.010**
	Female	GG	1.00 (reference)	
		CG	0.84 (0.69-1.02)	
		CC	0.58 (0.39-0.87)	
		C vs G	0.74 (0.57-0.95)	**0.019**
				
Tumor location	Extremities	GG	1.00 (reference)	
		CG	0.81 (0.66-1.00)	
		CC	0.56 (0.38-0.82)	
		C vs G	0.77 (0.66-0.90)	**0.001**
	Other	GG	1.00 (reference)	
		CG	0.85 (0.70-1.03)	
		CC	0.59 (0.37-0.95)	
		C vs G	0.79 (0.63-0.99)	**0.041**
				
Age	≥23.5	GG	1.00 (reference)	
		CG	0.84 (0.70-1.01)	
		CC	0.59 (0.42-0.82)	
		C vs G	0.79 (0.66-0.95)	**0.011**
	<23.5	GG	1.00 (reference)	
		CG	0.79 (0.61-1.03)	
		CC	0.56 (0.36-0.87)	
		C vs G	0.75 (0.59-0.95)	**0.020**

## DISCUSSION

Deeper understanding of lncRNAs and their role in carcinogenesis could possess a large number of potential clues for developing novel therapeutic agents for OS. In this two-stage, case-control study, we examined the relationship between HOTAIR variants and OS risk among Chinese population. We identified that people with C allele of SNP rs7958904 had a decreased risk of developing OS. SNP rs7958904 also has a genotype-specific effect on lncRNA HOTAIR expression. Our findings support the hypothesis that functional genetic variants influencing lncRNA expression may explain a part of OS genetic basis. To our knowledge, this is the first study to examine the association between HOTAIR variants and OS risk.

lncRNA HOTAIR has been widely explored as a functional lncRNA participating in multiple cancers [26]. In 2007, Rinn et al [27] first characterized one well-placed lncRNA in HOX clusters: this 2158 nucleotide RNA, termed HOTAIR, was localized to a regulatory boundary in the HOXC cluster and was expressed in both distal and posterior fibroblasts. This means HOTAIR lifts noncoding RNAs to new levels [28]. Since then, HOTAIR was evaluated as an oncogenic factor and could be used as a prognostic biomarker in different cancer type [29]. In a meta-analysis with 748 patients from 8 studies, Cai et al [30] showed that the patients with high HOTAIR expression level had a higher incidence compared with that in patients with low HOTAIR expression level. On the other hand, suppressed expression of HOTAIR inhibits the proliferation and tumorigenesis both *in vitro* and *in vivo* [31]. Except of these, HOTAIR was additionally reported to have significant influence on the proliferation, metastasis, EMT, and drug resistance in various human cancers [29]. Conclusively, understanding the biological roles of HOTAIR in different cancer types may help us to recruit this lncRNA as a diagnostic or predictive biomarker.

In current study, we determined C allele of HOTAIR rs7958904 could decrease the OS risk, as well as the expression level of HOTAIR (OR: 0.77; 95% CI: 0.67-0.90). These results was consistent with a previous study by Xue et al [18], which identified individuals with rs7958904 CC genotype had a significantly decreased risk of colorectal cancer in both Stage 1 and 2, compared with those carrying GG genotype (OR = 0.67, 95% CI = 0.51-0.87 in combined stage). Through *in silico* analysis, the secondary structure of HOTAIR was remarkably changed with rs7958904 G/C variant [18]. However, another SNP rs4759314, which was significantly associated with the increased gastric cancer risk by Du et al [17], was not replicated in current study.

In conclusion, we identified a SNP located in HOTAIR gene (rs7958904) that was significantly associated with decreased risk of OS in our two-stage, case-control study. Furthermore, *in vivo* studies found HOTAIR was notably up-regulated in OS tissues than in adjacent normal tissues, and subjects with the rs7958904 CC genotype had significantly lower HOTAIR RNA levels than other genotypes. Strength of this study includes large sample size, study design, and homogeneous participants. Larger prospective studies and further studies into the detailed biological mechanisms of HOTAIR SNPs are warranted to confirm our results.

## MATERIALS AND METHODS

### Subjects

Totally included in this study were 500 subjects diagnosed with OS, and 500 healthy controls in stage 1, as well as 400 OS patients and 400 healthy controls in an independent validation stage (stage 2). Pathological diagnosis was made by an experienced pathologist and double-checked by another pathologist. The healthy controls free from any cancer and matched by gender and age, were recruited when they were attending a routine examination. Blood samples (5 ml) were obtained from the subjects who participated in the study. A structured questionnaire was used to elicit detailed information on demographic factors. All specimens were handled and made anonymous according to the ethical and legal standards. This study was approved by the institutional Review Boards. Written informed consents were obtained according to the Declaration of Helsinki from both groups.

### SNP selection and genotyping

Genomic DNA was extracted from peripheral blood mononuclear cells using the QIAamp DNA whole blood kit (QIAGEN Inc., Valencia, CA, USA). The tagSNPs were selected basing on data of Han Chinese population (HCB data) of the HapMap Project (HapMap Rel 27, NCBI B36) covering 10232bp region (6232bp HOTAIR locus and 2kb upstream as well as 2kb downstream regions of the HOTAIR gene). Three htSNPs (rs4759314, rs7958904 and rs874945) were selected with Haploview version 4.2 software. SNP genotyping was performed by the Sequenom MassARRAY RS1000 while Sequenom Typer 4.0 Software was used to perform data management and analysis. The genotyping analysis was done blind as regards participants. To control the quality, the selected PCR-amplified DNA samples were examined by DNA sequencing to confirm genotyping results.

### Quantitative real-time RT-PCR analyses of HOTAIR

A total of 100 OS tissues were obtained from pre-treatment patients. RNA was extracted from the frozen tumor and corresponding normal tissues by standard methods using TRIzol reagent (Invitrogen, Carlsbad, CA, USA) according to the manufacturer's instructions. The expression of HOTAIR was determined by SYBR Green Assay and the levels was calculated relative to expression of β-actin by the 2^−ΔCt^ method. All assays were conducted by using the ABI 7900 HT system (Applied Biosystems, Foster City, CA, USA). All reactions were performed in triplicate.

### Statistical analyses

Comparisons between groups were made using the x^2^ test (nominal data) or Student's t test (continuous data). Hard-Weinberg analysis was performed by comparing the observed and expected genotype frequencies of HOTAIR using chi-square test. The odds ratios (OR), with 95 % confidence interval (CI), were calculated by an unconditional logistic regression model. We evaluated the log-additive (which assumes an additive effect of each copy of the minor

allele) inheritance models for each SNP in relationship to OS case status. Statistical analysis was performed using SPSS13.0 software package (SPSS Company, Chicago, IL, USA). *P* value < 0.05 was considered statistically significant.
